# The Role of Neutrophil-to-Lymphocyte Ratio and Right Ventricular Dysfunction in Indonesian Patients with COVID-19: A Retrospective Cohort Study

**DOI:** 10.3390/jcm14062051

**Published:** 2025-03-18

**Authors:** Raksheeth Agarwal, Stanislaus Ivanovich Krishnanda, Oliver Emmanuel Yausep, Raka Aldy Nugraha, Gatut Priyonugroho, Siti Hertine, Sony Hilal Wicaksono, Prima Almazini, Dian Zamroni, Hary Sakti Muliawan

**Affiliations:** 1 Faculty of Medicine, Universitas Indonesia, Jl. Salemba Raya No.6, Jakarta Pusat 10430, Indonesia; stanislaus.ivan.tan@gmail.com (S.I.K.); oliveremmanuel@hotmail.com (O.E.Y.); priyonugrohog@gmail.com (G.P.); sonyhw@live.com (S.H.W.); dr.primz@gmail.com (P.A.); dzamroni@gmail.com (D.Z.); 2 Department of Medicine, Jacobi Medical Center/Albert Einstein College of Medicine, 1400 Pelham Parkway South, Bronx, NY 10461, USA; 3 Department of Cardiology and Vascular Medicine, Universitas Indonesia Hospital, Jl. Prof. Bahder Djohan, Depok 16424, Indonesia; rakaaldynugraha@hotmail.com (R.A.N.); tineazhari@gmail.com (S.H.); 4 Department of Pulmonology, Universitas Indonesia Hospital, Jl. Prof. Bahder Djohan, Depok 16424, Indonesia

**Keywords:** neutrophil-to-lymphocyte ratio, right ventricular dysfunction, transthoracic echocardiography, COVID-19, prognosis

## Abstract

**Background/Objectives**: The clinical impact of neutrophil-to-lymphocyte ratio (NLR) and right ventricular (RV) dysfunction on clinical outcomes in COVID-19 remains understudied in the Indonesian population. This study aims to investigate their prognostic value in hospitalized Indonesian adults with COVID-19. **Methods**: A retrospective cohort study was conducted at a COVID-19 referral hospital in Indonesia. We included all consecutive adults hospitalized between April 2020 and April 2021 who underwent transthoracic echocardiography (TTE) during admission. Clinical information was extracted from electronic medical records. TTE variables were defined according to the American Society of Echocardiography criteria. Statistical analyses were performed using SPSS. Ethical approval was obtained from the Institutional Review Board of Universitas Indonesia (#2022-01-135). **Results**: A total of 488 patients were included in this study—29 with and 459 without RV dysfunction. The mean age of the population was 54.8, with 42% being female. An NLR >4.793 was considered elevated. Elevated NLR was independently associated with RV dysfunction (OR: 3.38, *p* = 0.02). Older age (HR: 1.02, *p* = 0.01), obesity (HR: 1.85, *p* < 0.01), chronic kidney disease (HR: 1.69, *p* = 0.01), high NLR (HR: 2.75, *p* < 0.001), and RV dysfunction (HR: 2.07, *p* = 0.02) independently increased the risk of 30-day mortality by multivariate Cox regression analysis. **Conclusions**: In adult Indonesian patients hospitalized with COVID-19, an elevated NLR was associated with RV dysfunction, and both of these parameters increased the risk of 30-day mortality. This retrospective cohort study highlights the prognostic importance of NLR and RV dysfunction in hospitalized COVID-19 patients, providing physicians with tools to identify high-risk patients.

## 1. Introduction

Coronavirus disease 2019 (COVID-19), caused by severe acute respiratory syndrome coronavirus 2 (SARS-CoV-2), was first identified in December 2019. Its clinical presentation ranges from asymptomatic infection to critical illness characterized by acute respiratory distress syndrome (ARDS), septic shock, and multi-organ failure. Several prognostic markers have been identified, including the neutrophil-to-lymphocyte ratio (NLR), an inflammatory marker associated with disease severity and mortality [1]. Additionally, cardiovascular comorbidities have been shown to predict worse clinical outcomes in COVID-19 patients [2]. Therefore, beyond NLR, assessing both pre-existing and de novo cardiovascular abnormalities may provide valuable prognostic information.

The American Society of Echocardiography (ASE) has highlighted the importance of echocardiographic evaluation in COVID-19 patients [3]. Studies have demonstrated that RV dilation and dysfunction are common in severe COVID-19, and are associated with increased mortality [4,5,6]. Transthoracic echocardiography (TTE) is a non-invasive and accessible tool for assessing cardiac structure and function, including RV performance. In a global study of 1216 COVID-19 patients, TTE findings influenced clinical management decisions in 33% of the patients, demonstrating the value of this modality [7].

Given the rising burden of cardiovascular disease in Indonesia [8], assessment of NLR and RV dysfunction via TTE could provide valuable prognostic information. However, the prognostic value of these markers remains unexplored in the Indonesian population. Such prognostic information is tremendously valuable as it would allow for risk stratification and efficient resource allocation, especially in low-resource settings. This retrospective study aims to evaluate the prognostic value of NLR and RV dysfunction in hospitalized Indonesian adults with confirmed COVID-19. Additionally, we seek to determine whether an association exists between these markers, which could provide mechanistic insights.

## 2. Materials and Methods

### 2.1. Study Population and Design

This single-center retrospective cohort study was conducted at *Universitas Indonesia* Hospital, a designated COVID-19 referral hospital in Indonesia. All consecutive hospitalized adult patients with confirmed COVID-19 who underwent TTE assessment between 3 April 2020 and 6 April 2021 were included in this study. A confirmed case of COVID-19 was defined by a positive real-time reverse-transcriptase polymerase chain reaction (RT-PCR) test for SARS-CoV-2. Patients under the age of 18 or with negative real-time RT-PCR results were excluded. This retrospective study was performed in line with the principles of the Declaration of Helsinki, and ethical approval was granted by the Institutional Review Board (IRB) of Universitas Indonesia Hospital (Ref: 2022-01-135).

### 2.2. Transthoracic Echocardiography

Patients with appropriate indications for TTE, such as a clinical suspicion of congestive heart failure, a history of coronary artery disease, and new onset dyspnea, underwent focused TTE assessments during hospitalization. The decision as to whether a TTE study was needed was made by a clinical cardiologist as a part of the multidisciplinary team. All TTE assessments were conducted by cardiologists experienced in this technique. In accordance with recommendations from the ASE [9], the TTE assessments were focused as necessary to aid management decisions, and other appropriate infection control and protective precautions were taken. All TTE findings were recorded on a standardized echocardiography reporting form. The following TTE parameters were extracted for this study: left atrial (LA) dilation, left ventricle (LV) hypertrophy, LV ejection fraction, LV systolic function, right ventricle (RV) function, wall motion abnormalities (WMAs), and LV diastolic function. LA dilation was defined as an anteroposterior LA diameter > 40 mm measured in the parasternal long-axis view, as an LA volume index > 34 mL/m^2^, or by visual estimation. LV measurements were made in the parasternal long-axis view; LV hypertrophy (LVH) was defined as an LV mass > 95 g/m^2^ for women and >115 g/m^2^ for men. LV ejection fraction (LVEF) was measured by Simpson’s biplane method, measured by the TEICHOLZ quantification method on M-Mode, or estimated visually when image quality did not allow for accurate quantification. LV systolic dysfunction was defined as an LVEF < 50%. RV dysfunction was defined by an abnormal tricuspid annular plane systolic excursion (TAPSE) (<17 mm) or RV dilation (RV basal diameter > 41 mm or visual estimation). LV diastolic dysfunction was defined according to the structural and functional criteria set by the ASE [10]. Any WMAs were also recorded.

### 2.3. Data Collection

All data were extracted from the hospital’s secure electronic medical records (EMR) system. The extracted data included demographic information, peak severity of COVID-19 during hospitalization, final outcome of disease (survival or death), and presence of comorbidities, including obesity, hypertension, diabetes, dyslipidemia, chronic obstructive pulmonary disease (COPD), chronic kidney disease (CKD), congestive heart failure (CHF), coronary artery disease (CAD), and malignancy. The severity of COVID-19 was graded as mild, moderate, severe, or critical, as defined by the National Institutes of Health. TTE parameters were extracted from the patients’ standardized echocardiography report forms, also available on the EMR system.

### 2.4. Clinical Outcomes and Statistical Analysis

To determine the cut-off value for elevated NLR, a Receiver Operating Characteristics (ROC) curve was made for prediction of severe–critical COVID-19 disease. Youden’s J statistic was used to determine the optimal cut-off value. The normality of continuous variables was assessed using the Shapiro–Wilk test. Continuous variables were compared using Student’s *t*-test when normally distributed, or the Mann–Whitney U-test when non-normally distributed. Categorical variables were compared using the chi-square test or Fisher’s exact test. Multivariate logistic regression analysis was conducted to determine the factors associated with RV dysfunction in the cohort. Data for 30-day survival were extracted from the EMR. Survival analysis was conducted by formulating the Kaplan–Meier curve and by univariate and multivariate Cox regression analyses. A *p*-value of <0.05 was considered statistically significant. All statistical analyses were conducted using IBM SPSS Statistics 26 (IBM Corporations, Armonk, NY, USA).

## 3. Results

A total of 488 patients met the inclusion criteria, of which 459 patients had no RV dysfunction and 29 patients had RV dysfunction. More than half of the entire cohort developed severe–critical COVID-19, and approximately a quarter did not survive beyond 30 days of admission. The full characteristics of the patients included in the study are presented in Table 1.

### 3.1. Defining an Elevated NLR

**Figure 1:**
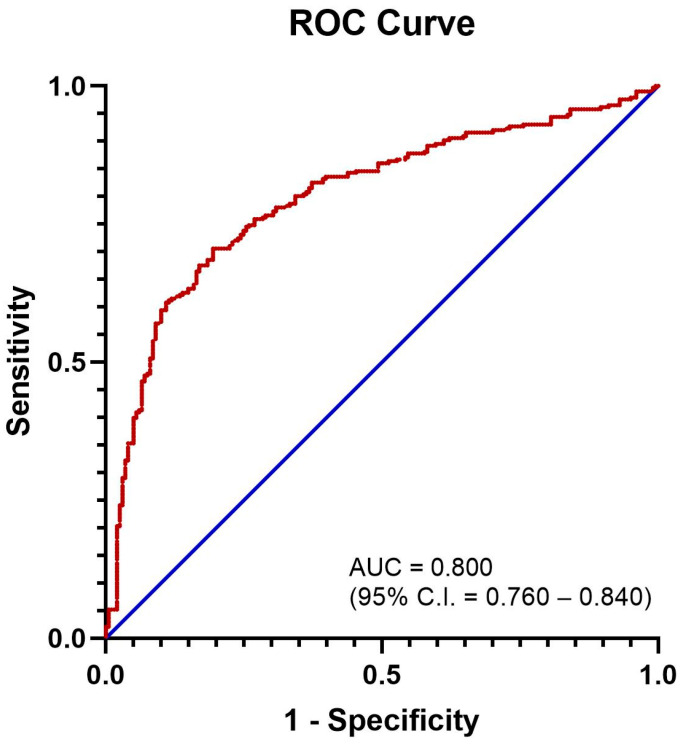
ROC curve for NLR in predicting severe–critical COVID-19. Area under curve = 0.800 (95% C.I. = 0.760–0.840).

Youden’s J index was calculated for each co-ordinate of the Receiver Operating Characteristics (ROC) curve (Figure 1). The co-ordinate with the highest J index was used to determine the optimal cut-off for the NLR, which was 4.793. This cut-off had a sensitivity of 70.6% and a specificity of 80.6% in predicting severe–critical COVID-19.

### 3.2. Factors Associated with RV Dysfunction

A logistic regression was performed to ascertain the effects of covariates on the likelihood that subjects have RV dysfunction (Table 2). The logistic regression model was statistically significant, χ^2^ = 64.48, *p* < 0.001. A high NLR (OR: 3.38, *p* = 0.02) and LV systolic dysfunction (OR: 9.76, *p* < 0.01) were independently associated with RV dysfunction.

### 3.3. Analysis of 30-Day Survival 

**Figure 2:**
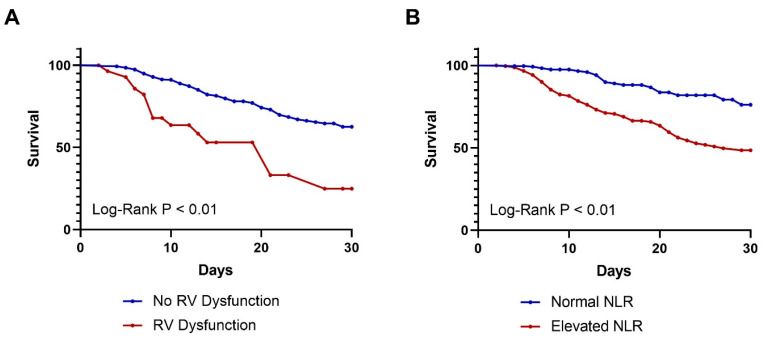
Kaplan–Meier 30-day survival curves by (**A**) presence of RV dysfunction (*p* < 0.01) and (**B**) presence of elevated NLR (*p* < 0.01).

The 30-day survival curves by presence of RV dysfunction and elevated NLR were plotted (Figure 2). Patients with RV dysfunction and an elevated NLR experienced lower rates of 30-day survival.

Next, a univariate Cox regression analysis was performed (Table 3). Older age (HR: 1.03, *p* < 0.001), obesity (HR: 1.71, *p* = 0.004), diabetes (HR: 1.85, *p* = 0.001), CKD (HR: 2.39, *p* < 0.001), CAD (HR: 1.57, *p* = 0.014), high NLR (HR: 3.38, *p* < 0.001), RV dysfunction (HR: 3.13, *p* < 0.001), and LV systolic dysfunction (HR: 1.08, *p* = 0.002) increased the risk of 30-day mortality.

Next, a multivariate Cox regression analysis was performed, incorporating the significant variables from the univariate analysis (Table 4). Older age (HR: 1.02, *p* = 0.01), obesity (HR: 1.85, *p* < 0.01), CKD (HR: 1.69, *p* = 0.01), high NLR (HR: 2.75, *p* < 0.001), and RV dysfunction (HR: 2.07, *p* = 0.017) independently increased the risk of 30-day mortality.

## 4. Discussion

Our study confirmed that both an elevated NLR and RV dysfunction are independently associated with mortality in COVID-19. We also showed that an elevated NLR is independently associated with RV dysfunction, highlighting the interplay between inflammation and cardiovascular dysfunction in this condition.

We observed that an elevated NLR nearly triples the risk of 30-day mortality (HR 2.75), which is consistent with the meta-analysis by Simadibrata et al. [1] and other studies from various geographical regions [11,12,13,14]. Previous Indonesian studies have also demonstrated that an elevated NLR is associated with disease severity in this population [15,16]. Severe COVID-19 is characterized by an exaggerated immune response and hyperinflammation, which drive disease progression [17]. This immune response leads to increased neutrophil production and recruitment, causing tissue damage via oxidative stress, elevated neutrophil elastase activity, and neutrophil extracellular trap (NET) formation [18]. Our study, along with others in the literature, reinforces that inflammation, as reflected by an elevated NLR, correlates with worse clinical outcomes in COVID-19.

Next, we found that RV dysfunction doubles the risk of mortality in COVID-19 patients (HR 2.07), which is consistent with prior studies [4,5,6,19]. Several mechanisms explain this association. First, severe COVID-19 often leads to acute respiratory distress syndrome (ARDS), increasing pulmonary vascular resistance (PVR) directly and through ventilatory strategies like positive-pressure ventilation [20]. Additionally, a well-known feature of COVID-19 is increased risk of venous thromboembolism (VTE), including pulmonary emboli, which contribute to PVR elevation and RV strain [6]. Elevated PVR and RV afterload results in distension of the highly compliant ‘afterload-sensitive’ RV, resulting in increased oxygen demand and reduced oxygen supply [20]. Additionally, direct myocardial damage from COVID-19 may contribute to RV dysfunction [6]. In critically ill patients, RV dysfunction is often the primary cause of cardiovascular insufficiency, resulting in worse outcomes. Furthermore, RV dilation may result in shifting of the septum to the left, impairing left ventricular (LV) filling and cardiac output [20].

Another important finding from our study is the independent association between elevated NLR and RV dysfunction. Patients with an elevated NLR had more than three times the odds (OR 3.38) of having RV dysfunction than those with a normal NLR. Inflammation can drive RV dysfunction in COVID-19 through various mechanisms. Systemic inflammation in COVID-19 triggers the release of pro-inflammatory cytokines (IL-6, TNF-α, IL-1β), impairing cardiac function directly and by promoting inflammatory cell infiltration [21]. Inflammation also causes endothelial dysfunction, leading to vasoconstriction, hypercoagulation (with increased risk of VTEs), and microvascular thrombi, all of which contribute to elevated PVR and RV strain. Furthermore, systemic inflammation exacerbates ARDS and pulmonary injury, compounding RV dysfunction.

Our study has various limitations that must be addressed. Firstly, the TTEs performed were focused, rather than comprehensive, to minimize the risk of infection. Secondly, the retrospective design introduces a higher risk of bias and reduced control over data availability. Thirdly, the timing of TTEs varied among patients, introducing variability in disease progression at the time of assessment. Abnormalities on TTE could develop at different stages of COVID-19 disease progression, and hence, the variability in TTE timing could confound the observed association between abnormal TTE findings and clinical outcomes. Fourthly, we did not differentiate patients by SARS-CoV-2 variants, and so the effect of different variants on TTE findings and clinical outcomes is unknown. Finally, since TTE was only performed once, we could not determine whether RV dysfunction was pre-existing or developed during hospitalization. Future studies should explore whether pre-existing RV dysfunction leads to inflammation, whether inflammation drives RV dysfunction, or whether the relationship is bidirectional. Nevertheless, our study aimed to evaluate the prognostic value of NLR and TTE findings during hospitalization, regardless of when abnormalities developed.

Despite these limitations, this was a real-world study on a large population, and hence provides important insights. If feasible and safe, TTE must be conducted in hospitalized COVID-19 patients with appropriate indications, as this non-invasive modality can provide valuable prognostic information. Together with abnormal inflammatory indices such as an elevated NLR, the presence of TTE abnormalities, especially RV dysfunction, can alert physicians to possible deterioration in patients. Future prospective studies should standardize the timing of measurements (e.g., within 72 h of admission) to improve consistency. Additionally, a risk scoring system could be developed, incorporating the five independent predictors of 30-day mortality from our study: older age, obesity, chronic kidney disease (CKD), high NLR, and RV dysfunction. This would better reflect real-world clinical scenarios where multiple risk factors interact.

## 5. Conclusions

In adult Indonesian patients hospitalized with COVID-19, an elevated NLR (>4.793) at admission and RV dysfunction independently increased the risk of 30-day mortality. There was an independent association between an elevated NLR and RV dysfunction in this population. These parameters can aid physicians in identifying high-risk patients early on in the course of their disease, and allocating resources accordingly.

## Figures and Tables

**Table 1:** Population characteristics.

	Entire Cohort (N = 488)		No RV Dysfunction (N = 459)		RV Dysfunction (N = 29)		*p*-Value
**Demographics and Past Medical History**							
Age, mean (SD)	54.82	13.50	54.34	13.50	62.32	11.19	0.005 ^†^
Males, N (%)	283	58.0%	261	56.9%	22	75.9%	0.044
Obesity, N (%)	225	46.1%	214	46.6%	11	37.9%	0.362
Hypertension, N (%)	326	66.8%	308	67.1%	18	62.1%	0.577
Diabetes, N (%)	188	38.5%	172	37.5%	16	55.2%	0.057
Dyslipidemia, N (%)	29	5.9%	28	6.1%	1	3.4%	1.000 ^‡^
COPD, N (%)	8	1.6%	6	1.3%	2	6.9%	0.076 ^‡^
CKD, N (%)	99	20.3%	90	19.6%	9	31.0%	0.138
CHF, N (%)	158	32.4%	140	30.5%	18	62.1%	<0.001
CAD, N (%)	149	30.5%	131	28.5%	18	62.1%	<0.000
Malignancy, N (%)	5	1.0%	4	0.9%	1	3.4%	0.265 ^‡^
**Hematological Indices and Other Echocardiographic Findings**							
NLR at admission, mean (SD)	6.85	6.33	6.52	5.91	11.93	9.82	<0.001 ^†^
Elevated NLR, N (%)	241	49.5%	218	47.6%	23	79.3%	0.001
LA dilation, N (%)	137	28.1%	124	27.0%	13	44.8%	0.038
LV systolic dysfunction, N (%)	34	7.0%	20	4.4%	14	48.3%	<0.001 ^‡^
LV diastolic dysfunction, N (%)	220	45.2%	210	45.9%	10	34.5%	0.233
**Admission outcomes**							
Severe COVID-19 disease, N (%)	286	58.6%	261	56.9%	25	86.2%	0.002
30-day mortality, N (%)	121	24.8%	105	22.9%	16	55.2%	<0.001
In-hospital mortality, N (%)	134	27.5%	118	25.7%	16	55.2%	0.001
Length of stay, mean (SD)	17.48	10.23	17.71	10.28	13.72	8.76	0.008 ^†^

RV = right ventricle. SD = standard deviation. COPD = chronic obstructive pulmonary disease. CKD = chronic kidney disease. CHF = congestive heart failure. CAD = coronary artery disease. NLR = neutrophil-to-lymphocyte ratio. LA = left atrium. LV = left ventricle. ^†^ Mann–Whitney U-Test. ^‡^ Fisher’s exact test.

**Table 2:** Logistic regression analysis for RV dysfunction.

Covariates	Odds Ratio	*p*-Value
Male sex	1.83 (0.65–5.14)	0.249
Age	1.04 (0.99–1.08)	0.094
Obesity	0.61 (0.24–1.55)	0.302
Hypertension	0.99 (0.36–2.72)	0.991
Diabetes	1.54 (0.62–3.81)	0.354
COPD	2.63 (0.27–25.20)	0.402
CKD	0.80 (0.29–2.23)	0.669
CHF	1.60 (0.61–4.24)	0.341
CAD	1.76 (0.69–4.51)	0.237
Dyslipidemia	0.66 (0.06–7.47)	0.736
High NLR	3.38 (1.19–9.59)	0.022
LA dilation	1.21 (0.44–3.32)	0.714
LV systolic dysfunction	9.76 (3.27–29.09)	<0.001
LV diastolic dysfunction	0.47 (0.18–1.24)	0.127

COPD = chronic obstructive pulmonary disease. CKD = chronic kidney disease. CHF = congestive heart failure. CAD = coronary artery disease. NLR = neutrophil-to-lymphocyte ratio. LA = left atrium. LV = left ventricle.

**Table 3:** Univariate Cox regression analysis (30-day survival).

Variables	Hazard Ratio	*p*-Value
Male sex	1.15 (0.80–1.65)	0.462
Age	1.03 (1.01–1.04)	<0.001
Obesity	1.71 (1.19–2.46)	0.004
Hypertension	0.94 (0.64–1.37)	0.742
Diabetes	1.85 (1.29–2.66)	0.001
COPD	0.84 (0.21–3.42)	0.812
CKD	2.39 (1.65–3.46)	<0.001
CHF	1.05 (0.72–1.53)	0.802
CAD	1.57 (1.09–2.26)	0.014
Malignancy	0.77 (0.11–5.48)	0.789
Dyslipidemia	0.92 (0.43–1.97)	0.821
High NLR	3.38 (2.18–5.22)	<0.001
RV dysfunction	3.13 (1.85–5.30)	<0.001
LA dilation	1.39 (0.96–2.03)	0.085
LV systolic dysfunction	2.38 (1.39–4.10)	0.002
LV diastolic dysfunction	1.08 (0.75–1.54)	0.691

COPD = chronic obstructive pulmonary disease. CKD = chronic kidney disease. CHF = congestive heart failure. CAD = coronary artery disease. NLR = neutrophil-to-lymphocyte ratio. RV = right ventricle. LA = left atrium. LV = left ventricle.

**Table 4:** Multivariate Cox regression analysis (30-day survival).

Variables	Hazard Ratio	*p*-Value
Age	1.02 (1.01–1.04)	0.010
Obesity	1.85 (1.28–2.67)	0.001
Diabetes	1.37 (0.95–1.98)	0.095
CKD	1.69 (1.13–2.52)	0.010
CAD	1.19 (0.81–1.75)	0.375
High NLR	2.75 (1.76–4.30)	<0.001
RV dysfunction	2.07 (1.14–3.76)	0.017
LV systolic dysfunction	1.07 (0.57–2.02)	0.830

CKD = chronic kidney disease. CAD = coronary artery disease. NLR = neutrophil-to-lymphocyte ratio. RV = right ventricle. LV = left ventricle.

## Data Availability

The data that support the findings of this study are available from the corresponding author upon reasonable request.
